# *Keratin 9* L164P mutation in a Chinese pedigree with epidermolytic palmoplantar keratoderma, cytokeratin analysis, and literature review

**DOI:** 10.1002/mgg3.977

**Published:** 2019-09-16

**Authors:** Xiaoliang Liu, Chuang Qiu, Rong He, Yuanyuan Zhang, Yanyan Zhao

**Affiliations:** ^1^ Department of Clinical Genetics Shengjing Hospital of China Medical University Shenyang China; ^2^ Department of Orthopaedics Shengjing Hospital of China Medical University Shenyang China

**Keywords:** cytokeratin, EPPK, intermediate filament, KRT9, mutation

## Abstract

**Background:**

Epidermolytic palmoplantar keratoderma (EPPK) is characterized by hyperkeratotic lesions on palms and soles. The disorder is caused by mutations of keratin 9 (*KRT9*) or *KRT1* gene.

**Methods:**

Epidermolytic palmoplantar keratoderma was diagnosed by physical examination and histopathological analysis in a five‐generation Chinese family. Mutation was screened by Sanger sequencing. The palmar expression of multiple cytokeratins were analyzed by tape‐stripping and Real‐time PCR. Literatures of EPPK with additional symptoms were reviewed.

**Results:**

Affected family members showed diffuse palmoplantar keratosis, with knuckle pads, friction‐related lesions and a novel additional symptom of palmar constriction. A heterozygous mutation of c.T491C (p.L164P) of *KRT9* was found within the helix initiation motif. The hydrophobic effect was decreased and the initiation of coiled‐coil conformation was delayed. The *KRT16*/*KRT6* expression were significantly increased in the patients, especially on the right, indicating activation of stress‐response and wound‐healing cytokeratins. There were also increased *KRT9*/*KRT2*, unchanged *KRT10*/*KRT1*, and undetectable *KRT14*/*KRT5* expression. The genetic and phenotypic heterogeneity of EPPK with additional symptoms were summarized by literature review.

**Conclusion:**

The p.L164P mutation of *KRT9* caused EPPK with a novel symptom of palmar constriction. The expression of multiple cytokeratins was altered in EPPK patients.

## 1 INTRODUCTION

Epidermolytic palmoplantar keratoderma (EPPK, OMIM 144200) is an autosomal dominant genodermatosis that manifests as hyperkeratosis confined to the palms and soles (Reis et al., 1994). The prime genetic candidate for EPPK is the keratin 9 (*KRT9*) gene on chromosome 17q21 wherein type I keratin gene cluster is located (Langbein, Heid, Moll, & Franke, 1993). KRT9 is exclusively expressed in the suprabasal layers of palmoplantar epidermis, and is essential for maintaining mechanical integrity of palmoplantar epidermis. It is structurally composed of a central rod α‐helical domain, flanked by non‐helical head and tail domains. Mutations of *KRT9* associated with EPPK usually occur in the helix initiation motif (HIM) or in the 1A rod domain close to HIM. Another less‐often candidate of EPPK is the *KRT1* gene belonging to type II keratin family on chromosome 12q13 (Hatsell et al., 2001).

Mutations of many EPPK cases are recurrent among different populations, yet the clinical manifestations are not the same. Some cases are associated with knuckle pads, nail lesions, and camptodactyly. We reported a novel symptom of palmar constriction in a Chinese EPPK pedigree in this study. We showed for the first time that heterozygous p.L164P mutation of *KRT9* associated with altered expression of multiple cytokeratins in EPPK patients.

## 2 MATERIALS AND METHODS

### 2.1 Patients

A five‐generation family with EPPK from Northeast China was included in this study. Peripheral blood samples were obtained from five affected and five unaffected family members. We followed the Declaration of Helsinki, and all protocols were approved by the Ethics Committee of Shengjing Hospital of China Medical University. All the participants were well informed, and consented for data publication in written.

### 2.2 Histopathologic analysis

Skin biopsy from the left palm of the proband was performed for histopathologic analysis. Photomicrographs were taken by OLYMPS IX51 inverted microscope (Olympus).

### 2.3 Mutation analysis

Genomic DNA from peripheral blood samples was isolated using the Blood Genomic DNA Miniprep Kit (Axygen). All the exons and the flanking splice junctions of *KRT9* were bidirectionally sequenced on ABI Prism 3730 Genetic Analyzer (Applied Biosystems). All primers in this study were designed using online software. The conservation and homology of sequences were assayed by the NCBI online resources (http://www.ncbi.nlm.nih.gov/). The protein secondary structure was analyzed by Expasy (http://www.expasy.org/).

### 2.4 Tape stripping and RNA isolation

Tape stripping was applied on the palms of five affected and five unaffected family members. Briefly, an area of 25 × 25 mm was repeatedly stripped for eight times by adhesive tapes (Sellotape GB Ltd.). Tapes were removed after a 2‐min adhesion with gentle pressure, rolled with the adhesive side out, and stored in individual tubes at −80°C until extraction. The stripping was performed three times with one‐month interval. The skin cells adherent to all tapes were pooled in one volume of buffer RLT and the RNA was isolated using RNeasy RNA extraction kit (Qiagen).

### 2.5 Reverse transcription and Real‐time PCR

Total RNA was reverse transcribed into cDNA using Sensiscript Reverse Transcriptase kit (Qiagen). Real‐time PCR was performed using the ABI 7900 System with all reagents purchased from Applied Biosystems. The expression of wild‐type and mutant *KRT9* mRNA was assayed by Taqman method, with probes FAM‐TTCTCGGC**T**GGCCTCTTAC‐MGB and VIC‐TTCTCGGC**C**GGCCTCTTAC‐MGB, respectively. The relative expression of *KRT9/KRT2, KRT16*/*KRT6*, *KRT10*/*KRT1*, and *KRT14*/*KRT5* were assayed by SYBR Green method. The *β‐actin* transcript was used as an internal control.

### 2.6 Statistical analysis

Statistical analysis was run using SPSS 16.0 software (SPSS). Data are presented by mean ± *SD*. Statistical differences were determined by independent‐sample *t* test. A value of *p* < .05 was considered significant.

## 3 RESULTS

### 3.1 Clinical findings

**Figure 1:**
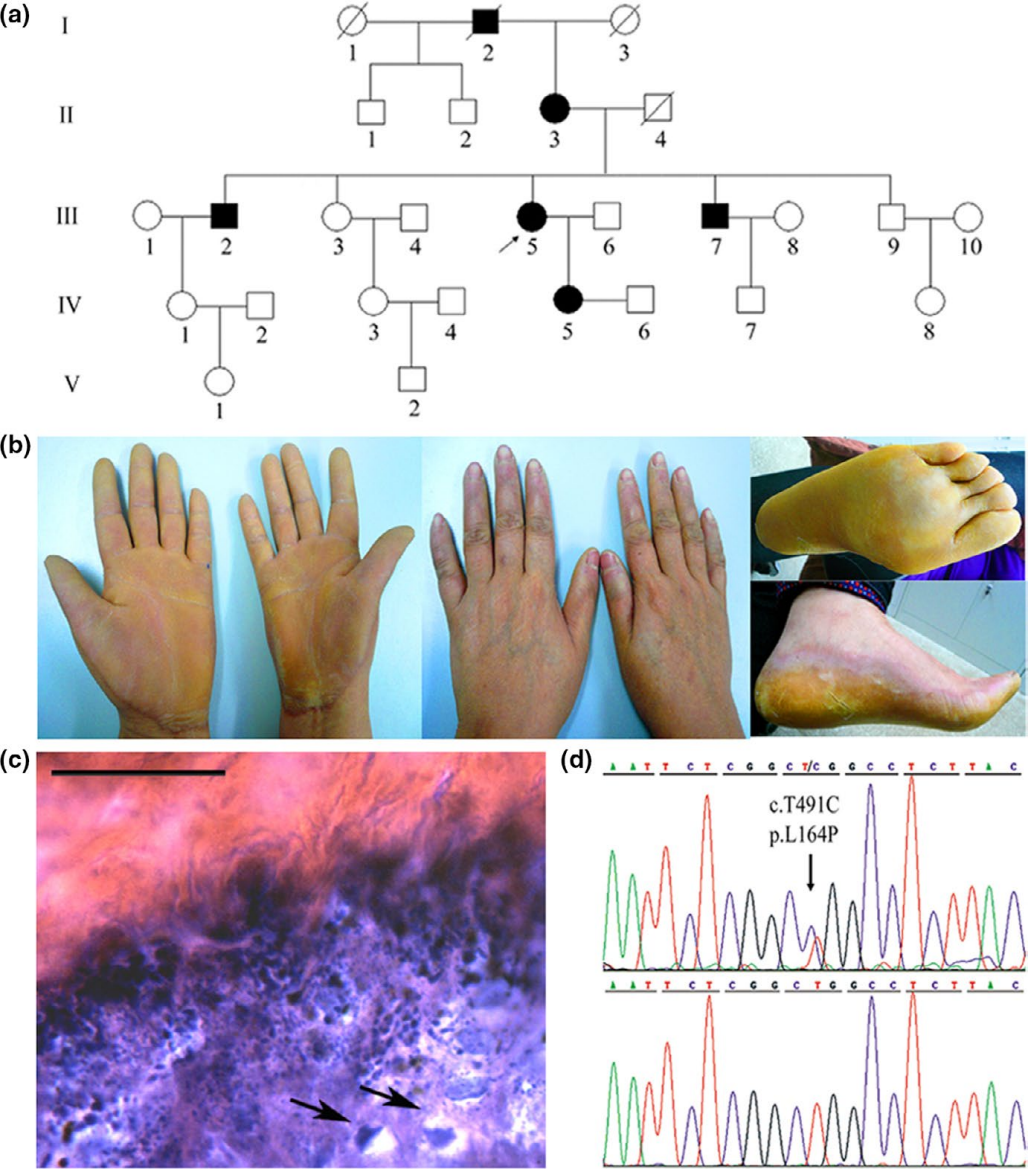
(a) Pedigree of the EPPK family. (b) Clinical presentation. There were diffuse yellowish thickening with erythematous margins on the palms and soles. Palm hyperkeratosis and knuckle pads were more severe on the right. The right palm was smaller than the left. (c) Histological presentation. The arrows indicate vacuolated cells. The bar represents 50 μm in image of ×400. (d) Sanger sequencing of the *KRT9* gene. The heterozygous variation in c.T491C (p.L164P) was found in the patient, not in the control

The hyperkeratosis was inherited in autosomal‐dominant pattern in the pedigree (Figure 1a). All affected individuals had similar symptoms. Epidermal hyperkeratosis started at age around 3 months. Diffuse yellowish thickening of the palmoplantar skin was surrounded by erythematous borders (Figure 1b). Knuckle pads were on the dorsal aspect of proximal interphalangeal joints. The hyperkeratosis and knuckle pads were more severe on the right, indicating friction‐related lesions. The frequently used right hands were irrationally smaller than the left. For the proband, the maximum width encompassing thenar muscles was 9.8 cm on the left and 8.1 cm on the right, indicating palmar constriction. There were mild decrease in heat and tactile sensitivity, slight difficulty in finger flexion, and fissuring on the feet. No other abnormalities were observed. Histopathologic analysis showed hyperplasia and hyperkeratosis, associated with hypergranulosis and acanthosis. Vacuolated cells were visible in the suprabasal layer (Figure 1c).

### 3.2 Mutation analysis

All exons of *KRT9* were sequenced, showing a heterozygous c.T491C (p.L164P) variation in patients but not in unaffected controls (Figure 1d). The mutation resides at the second *a* residue of the heptad repeats (*abcdefg*). It is highly conserved and homologous. The hydropathy index decreased from +3.8 of leucine to −1.6 of proline (Kyte & Doolittle, 1982). The protein secondary structure was assayed by Expasy, showing delayed initiation of the coiled‐coil conformation (Figure S1). We reviewed literature and the mutation was recurrent (Mao, Zhang, You, Xiao, & Zhao, 2018). We listed all the additional symptoms of EPPK with *KRT9* mutations in Table 1, with our data added in.

**Table 1:** Additional symptoms of EPPK with *KRT9* gene mutations

cDNA mutation^a^	Protein mutation^a^	Domain	Additional symptoms
c.A469G	p.M157V	1A	Blister (Hennies et al., 1994)
c.T470C	p.M157T	1A	Knuckle pad (Chen et al., 2009)
c.T470G	p.M157R	1A	Knuckle pad, camptodactyly and nails lesions (Liang et al., 2014)
c.C478T	p.L160F	1A	Knuckle pad and friction‐related lesion (Lu et al., 2003)
c.A481T	p.N161Y	1A	Cosegregate with breast and ovarian cancer (Torchard et al., 1994)
c.A482G	p.N161S	1A	Knuckle pad, (Li et al., 2019; Mao et al., 2018) hyperhidrosis and camptodactyly (Li et al., 2019)
c.A482T	p.N161I	1A	Knuckle pad and nail lesions (Küster et al., 2002)
c.C487T	p.R163W	1A	Knuckle pad, (Chiu et al., 2007; Codispoti et al., 2009; Lopez‐Valdez et al., 2013; Mao et al., 2018; Rothnagel et al., 1995; Xu et al., 2009) friction‐related lesion, (Chiu et al., 2007; Funakushi et al., 2009; Mayuzumi et al., 1999) blister, (Navsaria et al., 1995) parchment‐like scaling, (Warmuth et al., 2000) digital constriction, (Funakushi et al., 2009; Umegaki et al., 2011) camptodactyly (Lopez‐Valdez et al., 2013; Mao et al., 2018)
c.T491C	p.L164P	1A	Knuckle pad, palmar constriction, friction‐related lesions (this report)
c.T503C	p.L168S	1A	Knuckle pad (Li et al., 2009)
c.T1216C	p.C406R	2B	Knuckle pad (Wang et al., 2010)
c.C1282T	p.Q428X	2B	Knuckle pad (Umegaki et al., 2011)
c.T1373C	p.L458P	2B	Knuckle pad and camptodactyly (Du et al., 2011)

a Unified according to new version of *KRT9* sequence [GI:13653405].

### 3.3 Cytokeratin expression analysis

**Figure 2:**
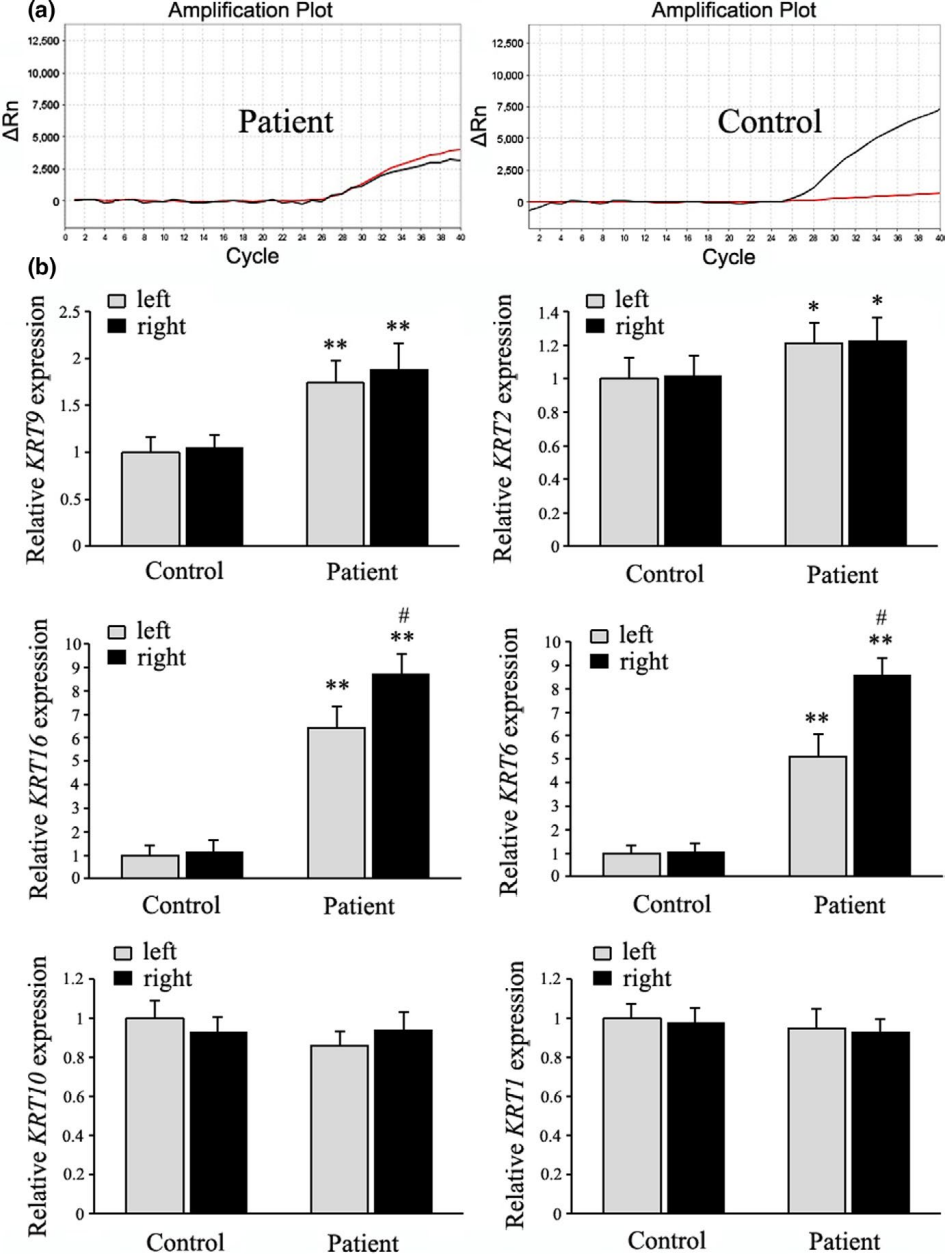
(a) Both wild‐type and mutant *KRT9* were detectable in the patient, not in the control by Taqman method of Real‐time PCR. (b) Relative expression of *KRT9/KRT2*, *KRT16*/*KRT6*, and *KRT10*/*KRT1* were assayed by quantitative Real‐time PCR, with the *β‐actin* mRNA as internal control. The bars are graphed as mean ± *SD* of fold over control left that is taken as “1.” Each sample was amplified in triplicates for at least three independent experiments. *: *p* < .05 compared with the control; **: *p* < .01 compared with the control; #: *p* < .01 compared with the left

Palmar epidermal cells were harvested non‐invasively by tape‐stripping. The total output of RNA was significantly higher in the patients than controls (left: 255.6 ± 49.2 vs. 28.3 ± 6.5 ng, *p* < .01; right: 276.8 ± 61.4 vs. 30.6 ± 6.2 ng, *p* < .01). Both wild‐type and mutant *KRT9* mRNA were detectable in the patients (Figure 2a). We furthermore analyzed the expression of keratin pairs *KRT9/KRT2*, *KRT16/KRT6*, *KRT10/KRT1,* and *KRT14/KRT5* (Figure 2b). The relative expression of *KRT9* was higher in the patients than controls (*p* < .01). There was also an increase of *KRT2*, the partner keratin of *KRT9* (*p* < .05). The stress‐activated *KRT16*/*KRT6* were obviously upregulated in the patients than controls (*p* < .01). Moreover, the right palms showed even higher levels of *KRT16*/*KRT6* than the left in the patients (*p* < .01). The expression levels of *KRT10*/*KRT1* were not changed. The expression of *KRT14*/*KRT5* were not detectable in both groups (data not shown). Overall, the *KRT9* p.L164P mutation altered the expression of multiple cytokeratins in the palmar epidermis.

## 4 DISCUSSION

Mutations of most EPPK cases are recurrent among different populations, yet the clinical manifestations are not always the same, not only in the severity of keratosis but also in additional symptoms. The present Chinese EPPK pedigree showed typical clinical and histopathological features. There were three additional symptoms: knuckle pads, friction‐related lesions and palmar constriction. By reviewing literature, 22 cases of EPPK with *KRT9* mutations showed additional symptoms. Knuckle pads happened in 15 cases and are the most common accompanying EPPK (68%). There were 11 *KRT9* mutations scattering from 1A to 2B domain for EPPK with knuckle pads, showing no specific mutation site (Chen, Xu, Cai, Chen, & Zhang, 2009; Chiu et al., 2007; Codispoti et al., 2009; Du et al., 2011; Küster, Reis, & Hennies, 2002; Li, Yang, Hua, Zhu, & Dai, 2009; Li et al., 2019; Liang, Liu, Huang, & Zeng, 2014; Lopez‐Valdez, Rivera‐Vega, Gonzalez‐Huerta, Cazarin, & Cuevas‐Covarrubias, 2013; Lu et al., 2003; Mao et al., 2018; Rothnagel et al., 1995; Umegaki et al., 2011; Wang, He, Song, Liu, & Chen, 2010; Xu, Chen, Chen, & Zhang, 2009). Ectopic expression of KRT9 was found in the pad, which might be responsible for the pad formation (Codispoti et al., 2009). Friction‐related lesions (18%) have been reported in Chinese, Indian, and Japanese patients with p.L160F and p.R163W mutations (Chiu et al., 2007; Funakushi, Mayuzumi, Sugimura, & Ikeda, 2009; Lu et al., 2003; Mayuzumi, Shigihara, Ikeda, & Ogawa, 1999). It is possible that the morbid epidermis might compensate for the decreased stability by overgrowing in response to mechanical friction, leading to hyperkeratosis in the frequently used hand. Palmar constriction is a novel finding, which is different from digital constriction in two Japanese patients with p.R163W mutation (Funakushi et al., 2009; Umegaki et al., 2011). Digital constriction occurred in the interphalangeal joints without hyperkeratosis, whereas palmar constriction occurred in the most hyperkeratotic regions. We are not sure whether the palmar constriction was due to merely reduced extension of the thick epidermal plaques, or to involvement of some deeper tissues. Other phenotypes have been reported in some studies such as camptodactyly (23%, Du et al., 2011; Li et al., 2019; Liang et al., 2014; Lopez‐Valdez et al., 2013; Mao et al., 2018), nail lesions (Küster et al., 2002; Liang et al., 2014), blister (Hennies, Zehender, Kunze, Küster, & Reis, 1994; Navsaria et al., 1995), scaling (Warmuth, Cserhalmi‐Friedman, Schneiderman, Grossman, & Christiano, 2000), hyperhidrosis (Li et al., 2019), and cosegregation with breast and ovarian cancer (Torchard et al., 1994). They were not found in our case. Overall, symptoms differ greatly even in cases carrying the same mutation. The diversity might lie in environmental or epigenetic factors, and needs further experiments on animals with specific *krt9* gene mutations.

Keratin family type I and II assemble to form heterodimers through coiled‐coil interaction of the rod domain composed by heptad repeats (*abcdefg*). Residues at position *a* and *d* stabilize dimer formation through hydrophobic effects (Langbein et al., 1993). The p.L164P at the second *a* position caused a great decrease in hydrophobic level, which should influence the formation and stability of heterodimers. Secondary structure analysis showed delayed initiation of the coiled‐coil conformation, indicating the mutation might be functional influencing.

Tape stripping is an effective and non‐invasive way to recover epidermal cells for RNA or protein assays (Wong et al., 2004). The *krt9*−/− mice had hyperpigmented calluses in the footpads and abnormal expression of many keratins (Fu et al., 2014). Hereby, the impact of *KRT9* p.L164P mutation on cytokeratin expression was analyzed by tape‐stripping and Real‐time PCR. The patients were more susceptible to loose cells and the RNA output was much higher. Both wild‐type and mutant *KRT9* were expressed in the patients. The mutation caused an increase in total abundance of *KRT9* and its partner *KRT2*. There was great induction of stress‐response and wound‐healing *KRT16*/*KRT6*, with even higher levels on the right, indicating activated signals of hyperproliferation in response to friction. *KRT10*/*KRT1* expressed predominantly in normal epidermis were not altered by the mutation. *KRT14*/*KRT5* expressed in undifferentiated cells of basal layer were not detectable, probably because tape‐stripping collected upper epidermis cells. Overall, the *KRT9* p.L164P mutation disturbed the expression of multiple cytokeratins.

In summary, we reported a Chinese EPPK family with a novel symptom of palmar constriction, which expanded the versatility of EPPK by literature review. We provided data about varied expression of cytokeratins in EPPK patients.

## CONFLICT OF INTEREST

The authors stated no conflict of interest.

## AUTHORS’ CONTRIBUTION

X.L. designed the experiment, analyzed the data, and drafted the manuscript. C.Q. acquired the clinical and pathological data. R.H. did the molecular experiments. Y.Zhang reviewed literature. Y.Zhao oversaw the study and revised the manuscript.
