# Supplementary material for: Keratin 9 L164P mutation in a Chinese pedigree with epidermolytic palmoplantar keratoderma, cytokeratin analysis, and literature review
Source: Mol Genet Genomic Med. 2019 Sep 16;7(11):e977. doi: 10.1002/mgg3.977 (PMC6825865; doi:10.1002/mgg3.977)
Supplement: Supplementary file 1 [file MGG3-7-e977-s001.doc]

**Supplementary Information**

***Keratin 9* L164P mutation in a Chinese pedigree with epidermolytic palmoplantar keratoderma, cytokeratin analysis and literature review**

**Xiaoliang Liu^1^ | Chuang Qiu^2^ | Rong He^1^ | Yuanyuan Zhang^1^ | Yanyan Zhao^1,*^**

^1^ Department of Clinical Genetics, Shengjing Hospital of China Medical University, Shenyang, China.

^2^ Department of Orthopaedics, Shengjing Hospital of China Medical University, Shenyang, China.

**^*^ Correspondence:** Yanyan Zhao. Department of Clinical Genetics, Shengjing Hospital of China Medical University, Shenyang, China. Email: [yyzhao@sj-hospital.org](mailto:yyzhao@sj-hospital.org)


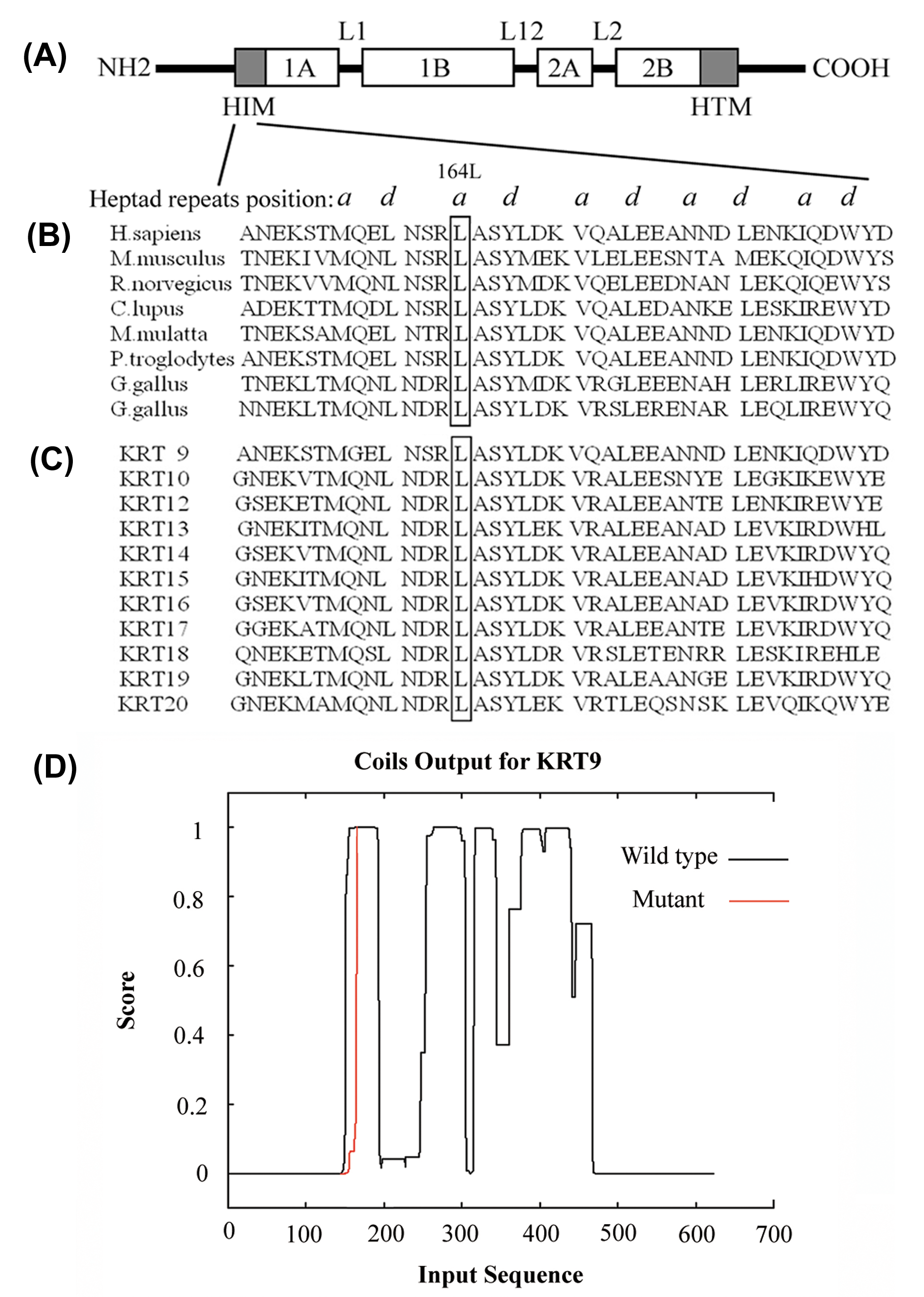


**FIGURE S1** (A) Schematic representation of *KRT9*. The 164 leucine was at *a* position of the heptad repeats in the helix initiation motif (HIM). (B) The 164 leucine of *KRT9* was conservative among different species. (C) The 164 leucine of *KRT9* was homologous among different keratin genes. (D) Secondary structure analysis of KRT9 protein by ExPASy. The initiation of the coiled-coil conformation of the mutant KRT9 (red) was delayed comparing with the wild type (black).
